# Enantioselective Aromatic Amination?

**DOI:** 10.1002/chem.202004151

**Published:** 2021-03-11

**Authors:** Vinzenz Thönnißen, Frederic W. Patureau

**Affiliations:** ^1^ Institute of Organic Chemistry RWTH Aachen University Landoltweg 1 52074 Aachen Germany

**Keywords:** atroposelective amination, Buchwald–Hartwig, Chan–Lam, enantioselective amination, Ullmann–Goldberg

## Abstract

The atroposelective formation of C−N bonds has recently emerged within the field of amination reactions. On first sight, it may seem quite surprising that such an ancient class of organic coupling reactions (Gabriel, Ullmann, Goldberg, Buchwald, Hartwig and many others) has so few enantioselective solutions, and this in spite of asymmetric synthesis being now a mature concept and field. Why should enantioselective C−N bond formation be so difficult? This question and some of the first examples that promise an imminent change of paradigm are herein discussed.

## Graphical abstract

**Direct C−N atroposelectivity**: Recent studies have demonstrated that a preexisting C−N bond is not necessary for the direct enantioselective construction of valuable scaffolds comprising a rotationally hindered aromatic C−N bond.

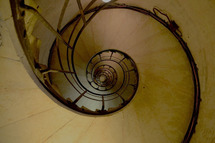

Chirality is usually associated with central chirality, but an important field of asymmetric synthesis would be atropoisomerism: the rotational hindrance around a usually single bond. In general, atropoisomerism is characterized by its rotational barrier (usually in kcal mol^−1^).^[1]^ A prominent example of this class of molecules is BINOL (2,2’‐binaphthol), but this kind of chirality is not limited to C−C biaryls. Indeed, atropoisomers with a rotationally hindered C−N bond are also important, and considerably more ubiquitous than often believed. Bioactive molecules such as, for example, Metolachlor or Ancistrocladinium A (Figure 1) are only a few examples for such atropoisomers.[^2^, ^3^, ^4^, ^5^] Strikingly however, C−N atropoisomerism is underrepresented as a research topic in the literature.^[6]^ This is due in part to the lack of general asymmetric methods for their construction, in spite of C−N bond formation and asymmetric synthesis being now mature fields, and in part to steric requirements for C−N atropostability.^[7]^

**Figure 1:**
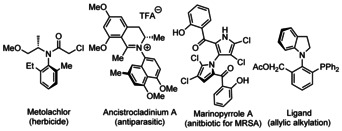
Important molecules with chiral C−N axis.

Evidently, surveying the recent literature indicates that atroposelective C−N bond formation is challenging. *Intermolecular* enantioselective aromatic C−N bond formation is even more so. Nevertheless, C−N atropoisomerism is not completely devoid of methodological solutions. For instance, an existing C−N bond can be post‐functionalized so as to atroposelectively increase steric congestion, by N‐arylation/alkylation,^[8]^ [2+2+2] cycloaddition,^[9]^ cyclisation,^[10]^ or halogenation.^[11]^ These early methods usually suffer however from limited substrate scope and complex catalytic systems. Most importantly, these early methods also avoid altogether the problem of asymmetric C−N bond *formation*, that is forming the C−N bond and imposing atroposelectivity at that bond in a concomitant event. Thus, the demand for general enantioselective aromatic amination and related methods is very high. The present concept article describes recent efforts towards the Grail in this field: that is the path towards enantioselective Gabriel, Ullmann–Goldberg, Chan–Lam, Buchwald–Hartwig,^[12]^ as well as oxidative and dehydrogenative amination reactions.^[13]^

Classically, the earliest methods to atroposelectively construct C−N bonds are diastereoselective: that is a chiral and often optically active substrate imposes atroposelectivity by intramolecular proximity with the reaction site (Scheme 1). For example, Kamikawa and Uemura reported a diastereoselective S_N_Ar reactions between a chiral planar organochromium complex and indoles.^[14]^ Moreover, Wencel–Delord and Colobert recently published a diastereoselective amination of chiral sufinyl hypervalent iodine reagents with indolines,^[15]^ and Yu introduced an oxidative amidation of N‐protected indoles with modified amino acids.^[16]^ These methods remain however limited to the concept of diastereoselectivity.

**Scheme 1:**
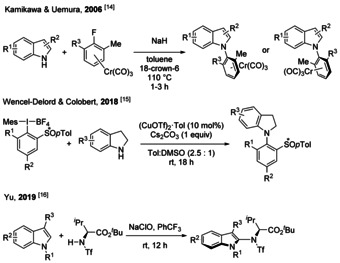
Diastereoselective amination and related reactions.

Unfortunately, classical amination reactions such as Buchwald–Hartwig or Ullmann–Goldberg, are ill suited for enantioselectivity due to generally harsh reaction conditions. Indeed, the essential reductive C−N bond formation step is a thermodynamically challenging process and often rate limiting. A promising lead to solve this general problem is certainly the development of novel chiral catalysts, whether organic or organometallic. These should improve reactivity by decreasing limiting activation barriers, as well as efficiently induce asymmetry. Some innovative and inspiring catalytic strategies are already being utilized for the enantioselective constructions of C−C biaryl atropoisomers. A few seminal examples include the coupling of quinones with phenols towards biphenols^[17]^ and azonaphthalene with heterocycles.^[18]^ Chiral phosphoric acids have been particularly successful as catalysts for the later enantioselective processes.

In aromatic enantioselective C−N bond forming series, one of the first breakthroughs is that of Bella and Jørgensen (2006, Scheme 2). Indeed, they and co‐authors published the presumed first direct enantioselective aromatic C−N bond forming reaction, utilizing 2‐naphthol derivatives as nucleophiles and azo dicarboxylates as preactivated electrophiles and internal oxidant. This reaction is catalyzed by a cinchona‐alkaloid and resulted in good *ee* (up to 98 %).^[19]^ It should be noted however that large functional groups were found necessary around the C−N axis for both configurational stability as well as efficient enantioselective induction. A related approach was developed by Zhang and co‐authors in 2019. They improved the stability of the atropoisomers by replacing 2‐naphthol derivatives by substituted 2‐naphthylamines.^[20]^ In the latter case the reaction was catalyzed by chiral phosphoric acids (CPA) and resulted in moderate to good *ee* (up to 93 %).

**Scheme 2:**
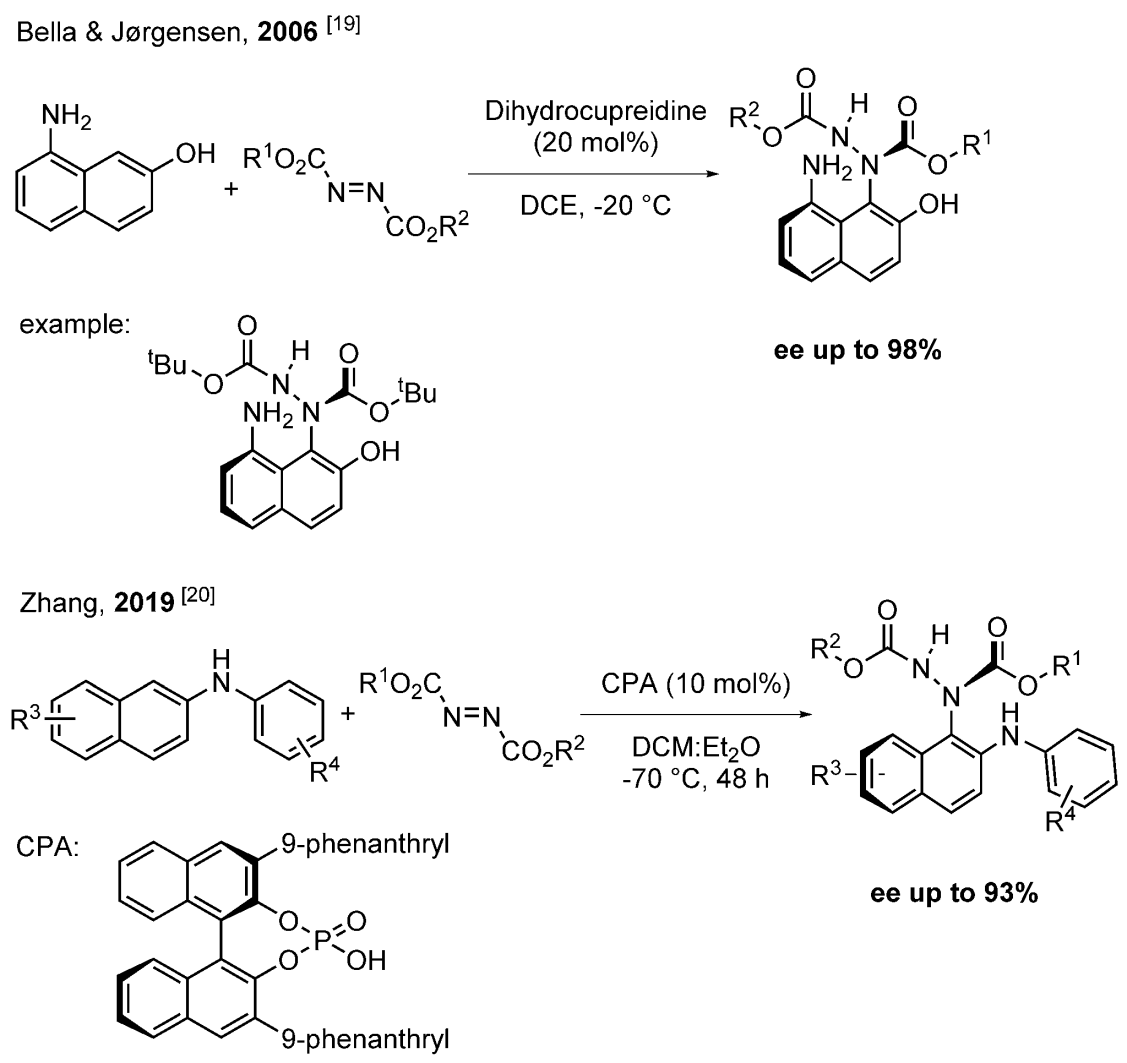
Enantioselective direct C−N bond forming reactions.

The next significant development in this field came most recently. In March 2020, a synthetic method from Li, Tan and co‐authors was published,^[21]^ introducing the redox neutral coupling of carbazoles and indoles as the nitrogen source with azonaphthalenes (Scheme 3). Because this reaction is redox neutral, no external oxidant is necessary. The phosphoric acid catalyst generates a chiral environment by hydrogen bonding. This transformation leads to good yields (51–97 %) and good to excellent *ee* (87–96 %). It must be noted however that only 2‐substituted carbazoles achieve high enantioselectivity. In addition, the configurational stability was investigated by heating a solution of the product (R^1^ = H, R^2^ = Me, R^3^ = *t*Bu, R^4^ = H) in toluene to 110 °C for 48 h. No difference of enantiopurity was thereafter observed. The substrate scope was moreover extended to include 3‐substituted indoles, achieving moderate to good yields (46–93 %) with excellent *ee* (92–99 %, Scheme 4).

**Scheme 3:**
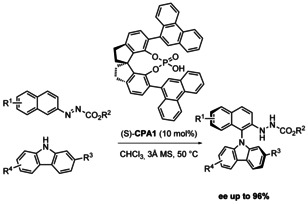
Li and Tan's enantioselective coupling of carbazoles with azonaphthalenes catalyzed by chiral phosphoric acids (2020).

**Scheme 4:**
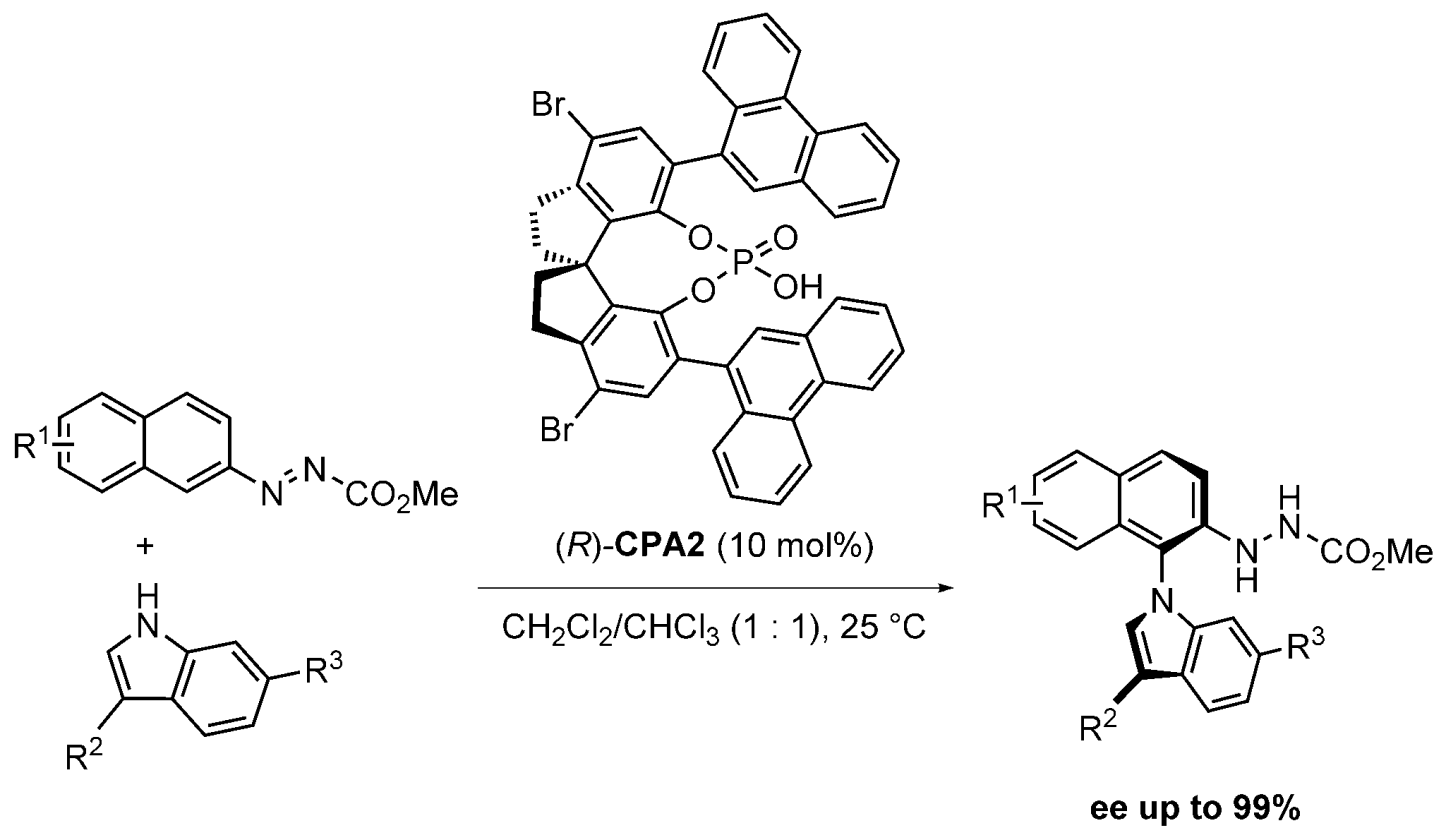
Li and Tan's enantioselective coupling of indoles with azonaphthalenes catalyzed by chiral phosphoric acids (2020).

Also very recently, Wencel–Delord and Colobert reported an enantioselective approach utilizing Csp^2^‐hypervalent iodine reagents as carbon substrates with 7‐substituded indolines as nitrogen substrates, under asymmetric copper catalyzed conditions (Scheme 5).^[22]^ The method may seem inconvenient at first sight in terms of hypervalent iodine prefunctionalization.

**Scheme 5:**
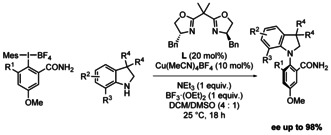
Wencel–Delord and Colobert's copper‐catalyzed enantioselective coupling of 7‐substituted indolines with hypervalent iodine reagents (2020).

Nevertheless, the latter method arguably constitutes a significant conceptual breakthrough, because it represents the first example of an enantioselective Ullmann–Goldberg‐type amination reaction. Indeed, while classical amination reactions such as Ullmann–Goldberg, Chan–Lam, Buchwald–Hartwig and others, require pre‐activation of the carbon substrate, those amination reactions are also very broad in scope as well as selective and functional group tolerant.^[12]^ For this reason, a general solution to the enantioselective aromatic amination problem will most probably have to come from the asymmetric derivatization of one or several of the above mentioned “name‐reactions”, by making the corresponding organometallic catalysts both more reactive and more enantioselective.

The substrate scope of that last method remains however limited at this point. Indeed, the C7 position is essential for an efficient enantioselective process (if R^3^ = H, e.r. = 47:53 only), highlighting again the importance of sterics not only for atropostability but also for high level asymmetric induction. Indeed, if substituted at C7 (R^3^ position), the enantiomeric ratio e.r. then ranges from 82:18 to 99:1, associated to promising yields (32 to 76 %).

In conclusion, the handful of recently published studies in the field of atroposelective aromatic amination and related C−N bond forming reactions promises new and general pathways for the enantioselective construction of carbon‐nitrogen bonds. The concept of C−N axial chirality and its properties are increasingly well understood,^[23]^ such that innovative methods and catalysts for their asymmetric construction should rise in the near future.

## Conflict of interest

The authors declare no conflict of interest.
